# Supplementary material for: Olfactory bulb differently synchronizes ventral hippocampus–medial prefrontal cortex circuit during spatial working memory across social dominance hierarchies
Source: PLoS One. 2026 Feb 12;21(2):e0341166. doi: 10.1371/journal.pone.0341166 (PMC12900306; doi:10.1371/journal.pone.0341166)
Supplement: S1 File — (a) Easy level of task (OB–mPFC), (b) Difficult level of task (OB–mPFC; ***p < 0.001, Values are expressed as mean ±SEM.), (c) Easy vs. difficult levels of task (OB–mPFC), (d) Easy level of task (OB–vHPC), (e) Difficult level of task (OB–vHPC), (f) Easy vs. difficult levels of task (OB–vHPC). (ZIP) [file pone.0341166.s001.zip › S1_File.pdf]

Cross-correlation Between OB Delta and mPFC Gamma Across Two Levels of Task Difficulty in Different Social Ranks

Cross-correlations were analyzed for *easy and difficult level of task*, and their comparison, considering correct vs. wrong responses in different social ranks, using two-way ANOVA.

Easy level of task:

No significant main effects of group ($F(2,162) = 2.34$, $p = 0.09$), response type ($F(1,162) = 0.74$, $p = 0.38$), or interaction ($F(2,162) = 0.61$, $p = 0.54$; Figure S1a) were found.

Difficult level of task:

Significant effects of group ($F(2,162) = 4.54$, $p < 0.001$) and interaction ($F(2,162) = 4.60$, $p < 0.001$) emerged, but not response type ($F(1,162) = 0.02$, $p = 0.86$; Figure S1b). Post-hoc Bonferroni tests showed higher delta–gamma correlation during wrong vs. correct responses in subordinates ($t(162) = 4.23$, $p < 0.001$). All three social ranks showed similar correlations during correct trials.

Easy vs. difficult levels of task:

There were no significant main effects of group ($F(2,162) = 0.20$, $P = 0.81$), response type ($F(1,162) = 0.35$, $P = 0.55$), and their interaction ($F(2,162) = 0.22$, $P=0.80$; Figure S1c).

Cross-correlation Between OB Delta and vHPC Gamma Across Two Levels of Task Difficulty in Different Social Ranks

Easy level of task:

No significant main effects of group ($F(2,162) = 2.30$, $p = 0.10$), response type ($F(1,162) = 1.09$, $p = 0.29$), or interaction ($F(2,162) = 0.99$, $p = 0.37$; Figure S1d).

Difficult level of task:

A significant interaction was found ($F(2,162) = 4.48$, $p < 0.05$), but neither group ($F(2,162) = 2.90$, $p = 0.05$) nor response type ($F(1,162) = 1.79$, $p = 0.18$) reached significance (Figure S1e),

Easy vs. difficult levels of task:

Significant group differences were observed (F (2,162) = 3.38, p < 0.05), but response type (F (1, 162) = 2.16, P = 0.11), and interaction (F (2,162) = 0.11, P = 0.73; Figure S1f) were not significant.

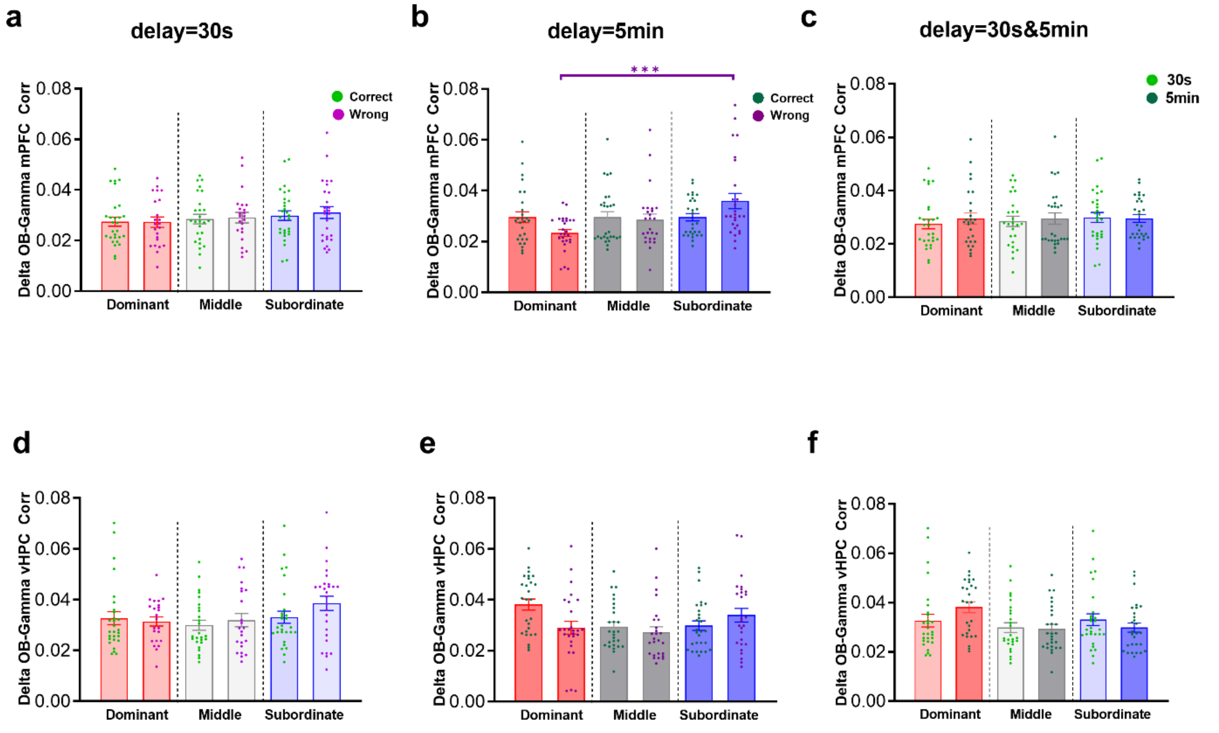

S1_Fig. Cross-correlation between OB delta and gamma oscillations in mPFC (a–c) and vHPC (d–f) during correct and wrong responses across two levels of task difficulty and different social ranks. (a) Easy level of task (OB–mPFC), (b) Difficult level of task (OB–mPFC; ***p < 0.001, Values are expressed as mean ±SEM.), (c) Easy vs. difficult levels of task (OB–mPFC), (d) Easy level of task (OB–vHPC), (e) Difficult level of task (OB–vHPC), (f) Easy vs. difficult levels of task (OB–vHPC).
