# Supplementary figures and images for: Olfactory bulb differently synchronizes ventral hippocampus–medial prefrontal cortex circuit during spatial working memory across social dominance hierarchies
Source: PLoS One. 2026 Feb 12;21(2):e0341166. doi: 10.1371/journal.pone.0341166 (PMC12900306; doi:10.1371/journal.pone.0341166)

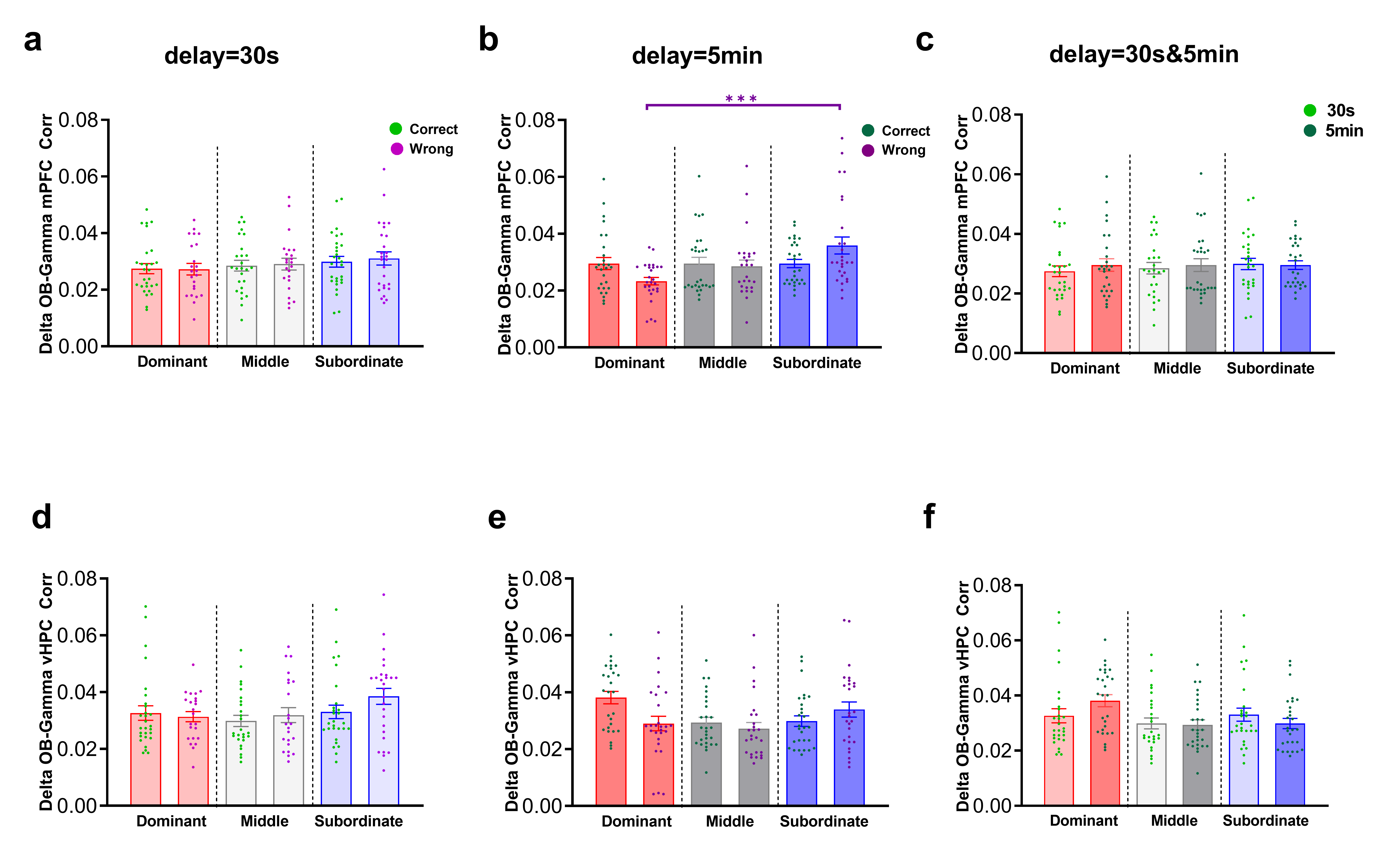

Supplement: S1 File — (a) Easy level of task (OB–mPFC), (b) Difficult level of task (OB–mPFC; ***p < 0.001, Values are expressed as mean ±SEM.), (c) Easy vs. difficult levels of task (OB–mPFC), (d) Easy level of task (OB–vHPC), (e) Difficult level of task (OB–vHPC), (f) Easy vs. difficult levels of task (OB–vHPC). (ZIP) [file pone.0341166.s001.zip › S1_Fig.tif]
