# Supplementary material for: Olfactory bulb differently synchronizes ventral hippocampus–medial prefrontal cortex circuit during spatial working memory across social dominance hierarchies
Source: PLoS One. 2026 Feb 12;21(2):e0341166. doi: 10.1371/journal.pone.0341166 (PMC12900306; doi:10.1371/journal.pone.0341166)
Supplement: Attachment — Submitted filename: Response to Reviewers.docx [file pone.0341166.s003.docx]

Manuscript Number.: **PONE-D-25-54057**

Olfactory Bulb Differently Synchronizes Ventral Hippocampus–Medial Prefrontal Cortex Circuit During Spatial Working Memory across Social Dominance Hierarchies

**Editor and Reviewer comments:**

We sincerely thank the editor and the reviewer for the time and thoughtful consideration they dedicated to evaluating our manuscript. We have carefully addressed all comments and revised the manuscript accordingly. We believe that the revisions made in response to their constructive feedback have significantly improved the quality and clarity of the work. Below, we provide a point-by-point response to each comment.

**#Journal Requirements:**

1. Please ensure that your manuscript meets PLOS ONE's style requirements, including those for file naming. The PLOS ONE style templates can be found at 1. Please ensure that your manuscript meets PLOS ONE's style requirements, including those for file naming. The PLOS ONE style templates can be found at

<https://journals.plos.org/plosone/s/file?id=wjVg/PLOSOne_formatting_sample_main_body.pdf> and [https://journals.plos.org/plosone/s/file?id=ba62/PLOSOne_formatting_sample_title_authors_affiliations.pdf](https://track.editorialmanager.com/CL0/https:%2F%2Fjournals.plos.org%2Fplosone%2Fs%2Ffile%3Fid=ba62%2FPLOSOne_formatting_sample_title_authors_affiliations.pdf/1/010f019ab3bec61f-385b7d4d-fd8b-461c-af19-1e0fad61ca9f-000000/a-IzMngs3ofYoMLtOoRWiEn0d5GwaeUfiWslyw6eLNM=238)
**Thank you for these clear guidelines. We have revised the manuscript to fully comply with PLOS ONE's style requirements. All changes are clearly indicated throughout manuscript.**

2. To comply with PLOS One submissions requirements, in your Methods section, please provide additional information regarding the experiments involving animals and ensure you have included details on (1) methods of sacrifice, (2) methods of anesthesia and/or analgesia, and (3) efforts to alleviate suffering.

**The Methods section has been updated to include further information on animal procedures, including the method of sacrifice, anesthesia and analgesia protocols, and measures taken to minimize animal distress. These additions are included in the “Electrode Implantation and Histological Verification” subsection.**

**#2.2.2.1. Electrode Implantation and Histological Verification: Anesthesia depth was continuously monitored by assessing tail and paw pinch reflexes. Additionally, vitamin A ointment was applied to the eyes to prevent corneal drying during surgery. Local anesthesia of the scalp was provided by subcutaneous injection of 0.5 ml Persocaine to minimize pain during the incision.**

**After the completion of experiments, to ensure accurate electrode placement, rats were deeply anesthetized with carbon dioxide. Once breathing ceased completely and the animals exhibited ocular pallor, they were rapidly decapitated using a guillotine. Brains were carefully extracted and fixed in 4% paraformaldehyde at 4 °C for 48 h. Subsequently, the brains were sectioned using a vibroslicer, stained with methylene blue, and examined under a microscope (AC 230V 50 Hz, Fig. 1b).**

3. When completing the data availability statement of the submission form, you indicated that you will make your data available on acceptance. We strongly recommend all authors decide on a data sharing plan before acceptance, as the process can be lengthy and hold up publication timelines. Please note that, though access restrictions are acceptable now, your entire data will need to be made freely accessible if your manuscript is accepted for publication. This policy applies to all data except where public deposition would breach compliance with the protocol approved by your research ethics board. If you are unable to adhere to our open data policy, please kindly revise your statement to explain your reasoning and we will seek the editor's input on an exemption. Please be assured that, once you have provided your new statement, the assessment of your exemption will not hold up the peer review process.

**Thank you for your comment. I would like to inform you that I have now made all the data publicly available in accordance with the journal’s open data policy.**

**#Data availability: The data associated with this research are openly accessible on the Open Science Framework (OSF) platform via the following link:**

<https://osf.io/sjex3/?view_only=c0da26cdab6e4141995620a703a016d8>

4. In the online submission form, you indicated that [Data will be made available upon reasonable request from the corresponding author.].

All PLOS journals now require all data underlying the findings described in their manuscript to be freely available to other researchers, either 1. In a public repository, 2. Within the manuscript itself, or 3. Uploaded as supplementary information.

This policy applies to all data except where public deposition would breach compliance with the protocol approved by your research ethics board. If your data cannot be made publicly available for ethical or legal reasons (e.g., public availability would compromise patient privacy), please explain your reasons on resubmission and your exemption request will be escalated for approval.

**In accordance with PLOS ONE policy, all relevant data have been deposited in the Open Science Framework (OSF) repository and are now publicly accessible.**

5. Please upload a new copy of Figure 1 as the detail is not clear. Please follow the link for more information: https://journals.plos.org/plosone/s/figures

**Thank you. A new, high-resolution version of Fig 1 has been uploaded according to the provided figure preparation guidelines.**

6. We notice that your supplementary figure (Figure S1) is uploaded with the file type 'Figure'. Please amend the file type to 'Supporting Information'. Please ensure that each Supporting Information file has a legend listed in the manuscript after the references list.

**Thank you for your helpful comment. We have amended the file type of Figure S1 to “S1_File” and uploaded it as a single ZIP file named “Supporting Information.” In addition, the corresponding legend in the manuscript has been updated as follows:**

**#Supporting Information: S1_File. Cross-correlation between OB delta and gamma oscillations in mPFC (a–c) and vHPC (d–f) during correct and wrong responses across two levels of task difficulty and different social ranks.** **(a) Easy level of task (OB–mPFC), (b) Difficult level of task (OB–mPFC; ***p < 0.001, Values are expressed as mean ±SEM.), (c) Easy vs. difficult levels of task (OB–mPFC), (d) Easy level of task (OB–vHPC), (e) Difficult level of task (OB–vHPC), (f) Easy vs. difficult levels of task (OB–vHPC).**

7. Please include captions for your Supporting Information files at the end of your manuscript, and update any in-text citations to match accordingly. Please see our Supporting Information guidelines for more information: <http://journals.plos.org/plosone/s/supporting-information>.

**Thank you for your guidance. We have prepared a 'Revised Manuscript with Track Changes' in which all modifications related to the Supporting Information captions and corresponding in-text citations are clearly highlighted.**

8. Please review your reference list to ensure that it is complete and correct. If you have cited papers that have been retracted, please include the rationale for doing so in the manuscript text, or remove these references and replace them with relevant current references. Any changes to the reference list should be mentioned in the rebuttal letter that accompanies your revised manuscript. If you need to cite a retracted article, indicate the article’s retracted status in the References list and also include a citation and full reference for the retraction notice.

**Thank you for your helpful recommendation. We have carefully reviewed the reference list and confirm that none of the cited articles have been retracted.**

**#Reviewer 1:**

1. The introduction is rather extensive for a scientific article, containing a detailed description of the current knowledge in the field. A significant reduction of the Introduction section is recommended

**Thank you for your valuable suggestion. We have carefully revised and shortened the Introduction section. All modifications related to this comment are clearly highlighted in the 'Revised Manuscript with Track Changes' for your reference.**

2. Attention should be given to the quality of the figures. The images in the PDF version of the manuscript appear to have low resolution.
**Thank you for your comment. We have replaced all figures with higher-resolution versions to ensure clear visibility in the PDF version of the manuscript.**
3. Throughout the text, the formatting of statistical values should be standardized. For example, ensure consistency between 'P=0.005' and 'P = 0.005’

**Thank you for your helpful comment. We have standardized the formatting of all statistical values throughout the manuscript. All corresponding edits can be seen in the 'Revised Manuscript with Track Changes'.**
